# The Spatial Distribution of the Japanese Beetle, *Popillia japonica*, in Soybean Fields

**DOI:** 10.1673/031.013.3601

**Published:** 2013-04-19

**Authors:** Stacey A. Sara, Emily B. McCallen, Paul V. Switzer

**Affiliations:** Department of Biological Sciences, Eastern Illinois University, 600 Lincoln Avenue, Charleston, IL 61920

**Keywords:** aggregation, edge effect, hedgerow, wind, sun

## Abstract

The Japanese beetle, *Popillia japonica* Newman (Coleoptera: Scarabaeidae), is a serious pest of many agricultural and horticultural plants. Relatively little research has investigated the distributions of Japanese beetles in agricultural fields, and this lack of information makes pest management more difficult. In the present study, the spatial distribution of Japanese beetles in soybean fields was examined. Specifically, how the distribution and abundance of beetles was affected by distance from an edge, edge direction, and edge type was examined. An edge effect for density was discovered; beetle numbers decreased significantly with increasing distance from the field edge. The east and south sides averaged higher numbers of beetles than the north and west. Downwind edges, in particular downwind edges adjacent to hedgerows, also had significantly higher beetle densities. In addition, females relatively far from the edge had larger egg loads than those closer to the edge. Differences in aggregation seeking behavior, in combination with movement in relation to wind and obstructions such as hedgerows, are possible explanations for these spatial patterns.

## Introduction

Population densities can change from the interior of a habitat to the edge of a habitat, resulting in an edge effect (e.g. Kunin 1998; Ries and Sisk 2004; Olson and Andow 2008). In insects, these edge effects could be caused by a variety of factors including host plant distributions, oviposition sites, predator or natural enemy distributions, microclimates, and windbreaks (McKone et al. 2001; Thomas et al. 2001; Haynes and Cronin 2006; Olson and Andow 2008; Luna and Xue 2009). Edge effects may exist for insect populations in agricultural fields (Winder et al. 1999; Olson and Andow 2008; Luna and Xue 2009). For instance, agricultural fields adjacent to a windbreak (e.g., a hedgerow) may create edge effects via the creation of a sheltered area, or because they act as a barrier to dispersal (Lewis 1970; Lewis and Dibley 1970; Thomas et al. 2001). Understanding such spatial patterns in agricultural fields can be important for developing effective management programs for insect pests (Winder et al. 1999; Thomas et al. 2001).

The Japanese beetle, *Popillia japonica* Newman (Coleoptera: Scarabaeidae), is an introduced pest in the United States and causes extensive damage to a variety of agricultural and ornamental plants (Fleming 1972). Adult *P. japonica* form aggregations on food plants, often defoliating the entire plant and causing economic loss (Fleming 1972; USDA/APHIS 2000). *P. japonica* distributions may be affected by factors such as host and non-host plant distributions (Bohlen and Barrett 1990; Holmes and Barrett 1997), plant height (Potter et al. 1996; Rowe and Potter 1996), sun (Rowe and Potter 2000), and wind (Fleming 1972).

Despite relatively little research on spatial distributions of *P. japonica*, edge effects in this species in agricultural fields have been observed. Gould (1963) anecdotally noted higher densities on soybean field edges adjacent to cornfields. Bohlen and Barrett (1990) documented higher *P. japonica* densities on the north side of agricultural fields, and attributed this pattern to prevailing southwest winds. Regniere et al. (1983) stated that aggregations on corn and soybean field edges form due to their proximity to beetles shifting locations.

In this study, the spatial distribution of *P. japonica* adults in soybean (*Glycine max*) fields was investigated. The primary objective of this study was to determine whether the density of *P. japonica* changed from the edge to the interior of a field. Although limited by the relative consistent wind direction during the study, possible wind and hedgerow effects on beetle densities were also investigated.

## Methods and Materials

A total of twenty-five soybean fields were each sampled once between 26 July 2009 and 10 August 2009 in Coles County and Edgar County, Illinois. Aerial maps were used initially to identify fields with hedgerows and the type of habitat bordering the field edges. To minimize variability for this study, acceptable edge types were limited to cornfield, soybean field, hedgerow, or road. Hedgerows were approximately 10 to 15 m high and consisted of tall trees mixed with shrubs and weeds. Only fields with straight edges running north to south and east to west were used, and each field had one or two hedgerow edges. The average field size was 22 ± 2.4 hectares, and to our knowledge these fields were not sprayed for beetles immediately prior to sampling.

We sampled each field between 07:00 and 18:00. The fields were sampled after the soybeans were large enough to have developed full canopies, meaning there was no space between crop rows at the top of the plants. A total of seventeen transects were sampled per field, each 30 m long and a half-meter wide. On each side of the field, four transects were sampled parallel to the edge at 1, 5, 10, and 25 m from the edge. These transects were located at the center of each side of the field, away from field corners. The final transect was located in the center of the field, parallel with the soybean rows. For each transect, the number of beetles present was recorded. A sample of single and paired beetles was collected, with a maximum of 10 single beetles and 10 pairs of beetles collected per transect, for later analysis of egg load. On the day of sampling, whether or not host plants were in the adjacent hedgerow was recorded, and beetle presence and damage in the hedgerow for 50 m, centered on the transect location, was noted. Potential host species recorded in the hedgerows included multiflora rose (*Rosa multiflora*), sassafras (*Sassafras albidum*), hackberry (*Celtis occidentalis*), primrose (*Oenothera biennis* L.), and buckwheat (*Fallopia scandens*).

### Analyses

The collected beetles were frozen for later analysis. Sex was determined by using foreleg morphology (Smith and Hadley 1926). Each female was dissected, and her mature eggs were counted (Saeki et al. 2005).

All statistical analyses were done using SAS version 9.1 (SAS Institute, www.sas.com) or Statview version 5.0 (SAS Institute). There was significant variation in total beetle number and egg loads between fields. To account for this variation while still making field comparisons, transect residuals were analyzed.

That is, the mean value per transect was determined for each field, and then the residual values for each transect were calculated by subtracting the mean value of the field from each transect value. ANOVA analyses was used to analyze the effects of side (north, east, south, or west) and transect distance from edge on the residuals of total beetle number per transect. Separate ANOVA analyses were done within each side on edge type (hedgerow, corn, or road) due to unequal sample sizes among edge types and to eliminate any effect of side on the values. The Tukey-Kramer test was used to analyze all post-hoc pair-wise comparisons. Because egg loads deviated from normality, the Kruskal-Wallis and Spearman nonparametric tests were used when investigating possible egg load patterns. All means are reported ± SE.

For wind analyses, wind data was obtained from the weather station at Eastern Illinois University (as reported by www.wunderground.com), which is within 28 km of all fields. Wind speed and direction were recorded approximately every 10 minutes. It was assumed that any effect of wind would be cumulative (i.e., not instantaneous) and occur during the period when the beetles are most active, which is between 09:00 and 15:00 (Fleming 1972). Therefore, the wind directions were converted to degrees, and the directions were averaged using circular statistics for this active period (Zar 1996). The average wind direction of the previous active period was then used for each field. If a field was sampled before noon, the previous day's wind direction was used for analysis. For fields sampled after 13:00, the wind direction from that day was used for analysis.

To analyze the effect of wind, each field edge was classified as downwind or upwind from the average direction calculated for that field.

**Figure 1.**
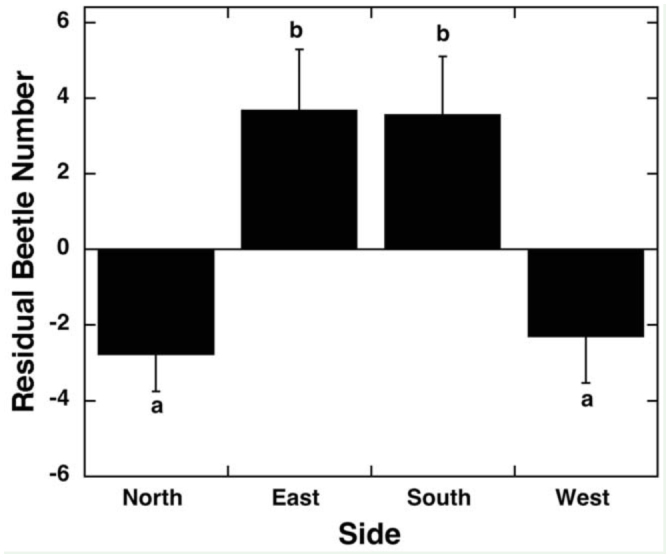
Average (± SE) residual of total number of *Popillia japonica* per transect for north sides (N = 100), east sides (N = 100), south sides (N = 100), and west sides (N = 100) of 25 soybean fields. Different letters (a, b) indicate a difference at the *p* < 0.05 level in post hoc comparisons. High quality figures are available online.

For instance, a southwest wind would make the south and west sides of the field upwind from the north and east sides. Fields with parallel edges to wind direction (e.g., north and south edges, which run parallel with a wind from the west) were eliminated due to low sample sizes. Three wind directions occurred during this study: northeast, northwest, and southwest. The average wind speed was 4.10 ± 0.14 m/sec.

To investigate possible effects of nearby host plants and/or beetle damage on beetle density in the field, beetle numbers for the 1-m and 5-m transects adjacent to hedgerows were combined. An ANOVA was then used to compare the total beetle number in adjacent transects among hedgerows with i) host plants and feeding damage, ii) host plants without feeding damage, and iii) no host plants.

## Results

### Total beetle number

**Figure 2.**
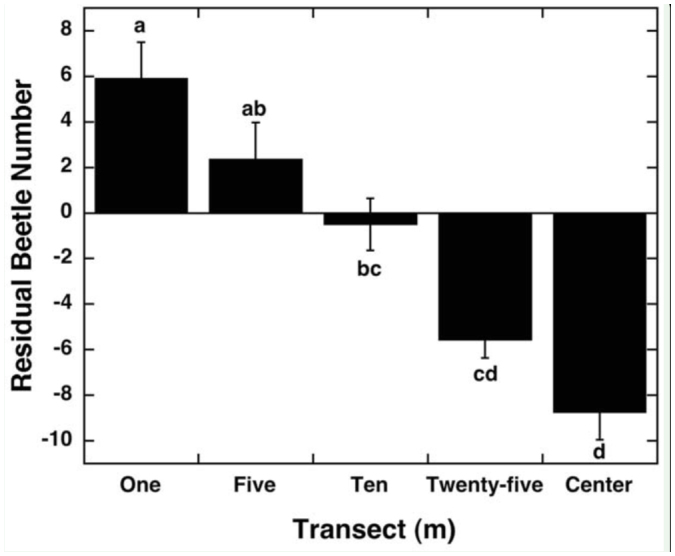
Average (± SE) residual of total beetle number per transect at varying distances from each field edge; 1-m transects (N = 100), 5-m transects (N = 100), 10-m transects (N = 100), 25-m transects (N = 100), and center transects (N = 25) of 25 soybean fields. Different letters (a, b, c) indicate a difference at the *p* < 0.05 level in post hoc comparisons. High quality figures are available online.

There were significant differences in beetle number residuals for both side and distance from edge. The south and east sides averaged significantly more beetles than the north and west sides (Figure 1; R^2^ = 0.17, F_3,384_ = 7.40, *p* < 0.0001). Beetle numbers were highest in the 1-m and 5-m transects, and decreased with distance from edge (Figure 2; R^2^ = 0.17, F_3,38_4 = 13.66, *p* < 0.0001). The interaction of side and distance from edge was not statistically significant. There were no differences among edge types within the north, east, and south sides (R^2^ < 0.04, F_2,89_ < 1.2, *p* > 0.2). However, the west side had a significant difference between edges adjacent to corn fields and hedgerows (average residuals: corn: 1.63 ± 1.96 beetles, N = 44; hedgerow: -5.35 ± 1.95 beetles, N = 32; road: -6.50 ± 3.46 beetles, N = 16; R^2^ = 0.08, F_2,89_ = 4.04, *p* = 0.02).

**Figure 3.**
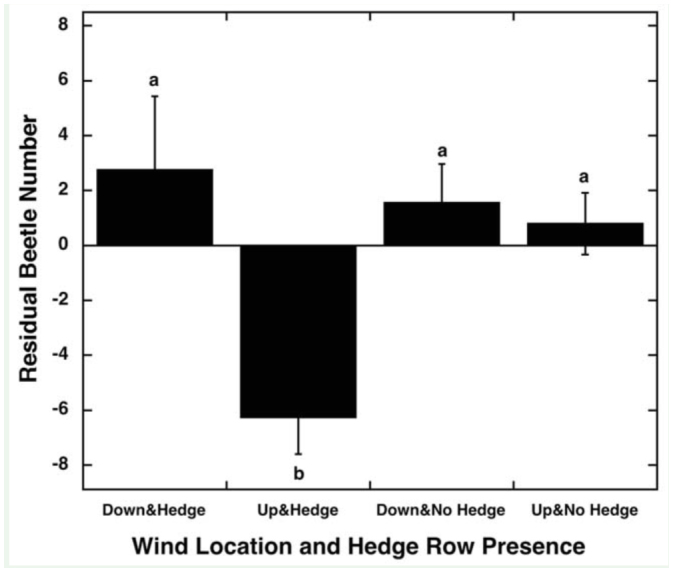
Average (± SE) number of *Popillia japonica* residuals per transect for downwind hedgerow edges (N = 60), upwind hedgerow edges (N = 36), downwind non-hedgerow edges (N = 80), and upwind non-hedgerow edges (N = 112) for 18 soybean fields. Different letters (a, b) indicate a difference at the *p* < 0.05 level in post hoc comparisons. High quality figures are available online.

The analysis of wind on beetle density revealed a significant difference between the downwind and upwind edge locations (R^2^ = 0.14, F_1,272_ = 7.81, *p* = 0.006) and a significant interaction of location and hedgerow presence (Figure 3; R^2^ = 0.14, F_1,272_ = 5.58, *p* = 0.019). The hedgerow presence alone had no significant effect (R^2^ = 0.14, F1,272 = 2.8, *p* = 0.10); however, the upwind hedgerow edges had significantly fewer beetles than the downwind hedgerow edges, downwind non-hedgerow edges, and the upwind non-hedgerow edges (Figure 3).

Adjacent plants did not seem to have an effect on beetle presence. Beetle density in the 1-m and 5-m transects was not significantly different among hedgerows with host plants and beetle feeding damage, hedgerows with host plants without feeding damage, and hedgerows without host plants (average residuals: host plants with damage: 1.82 ± 2.99 beetles, N = 38; host plants without damage: -2.09 ± 1.63 beetles, N = 8; no host plants: 13.87 ± 7.67 beetles, N = 18; R^2^ = 0.07, F_2,61_ = 2.24, *p* = 0.12).

### Egg load

**Figure 4.**
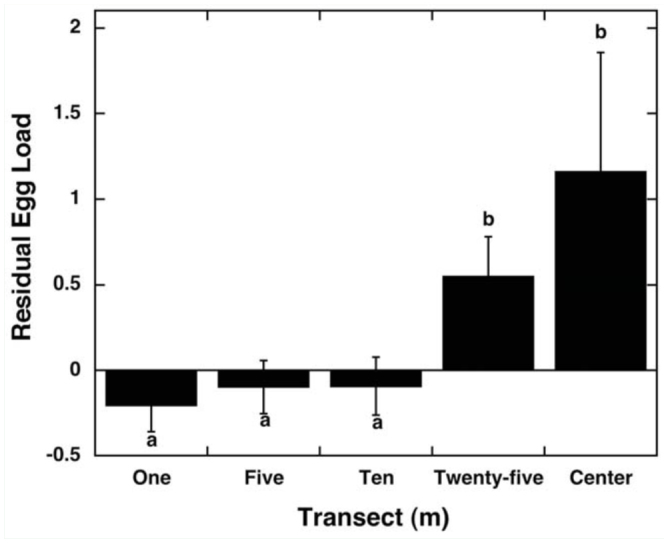
Average (± SE) residual egg loads for female *Popillia japonica* in 1-m transects (N = 481), 5-m transects (N = 410), 10-m transects (N = 347), 25-m transects (N = 216), and center transects (N = 49) collected in 25 soybean fields. Different letters (a, b) indicate difference at the *p* < 0.05 level in post hoc comparisons. High quality figures are available online.

Egg load showed significant differences among transect distances, with egg loads increasing with increasing distance from edges (Figure 4; Kruskal-Wallis: χ^2^_4,4625_ = 12.81, *p* = 0.012). Egg load did not differ among edge types or sides (edge: Kruskal-Wallis: χ^2^_3,1576_ = 2.88, *p* = 0.41; side: Kruskal-Wallis: χ^2^_3,1576_ = 6.16, *p* = 0.10).

## Discussion

Beetles were not evenly distributed throughout the fields; the highest densities occurred near field edges, especially on edges downwind and adjacent to a hedgerow. Egg load was highest for females away from the edge of the field. These results suggest that both population and individual characteristics of *P. japonica* were influenced by distance from habitat edge, the surrounding environment, and wind. These influences are discussed in more detail below.

### Total beetle number

Higher numbers of beetles were present on field edges. Regniere et al. (1983) suggested that aggregations of Japanese beetles on soybean field and cornfield are temporary as the beetles move from one feeding site to another. However, for the present study, a more likely explanation for the higher densities on edges could be that the beetles were responding to the abrupt change in habitat. Bohlen and Barrett (1990) and Holmes and Barrett (1997) found that *P. japonica* dispersal was reduced by non-host patches of habitat, and this pattern could cause an accumulation of beetles at the edge.

In addition to having higher densities at field edges, beetle density was also higher on the east and south sides of the soybean fields. Bohlen and Barrett (1990) noted a directional pattern in *P. japonica* distribution; however, in their study, the north side had a higher density than the south, and they attributed this pattern to southwest winds. As in Bohlen and Barrett's (1990) study, the present study found higher beetle numbers on the downwind sides than on the upwind sides. *P. japonica* uses olfaction to detect host plant volatiles (Fleming 1972; Ahmand 1982) and kairomones caused by feeding aggregations (Loughrin et al. 1995, 1996). When olfactory stimuli are present, the beetles will fly upwind towards the stimuli (Smith and Hadley 1926; Hawley and Metzger 1940; Fleming 1972). Beetles flying upwind towards olfactory stimuli may stop when the target is reached, forming or joining feeding aggregations on the downwind edges. In the absence of olfactory stimuli, *P. japonica* will fly with the wind (Fox 1927; Fleming 1972). When flying downwind, the beetles may respond to the abrupt change in habitat and drop out of the wind stream at the downwind edge of the soybean field.

Some evidence was found that the edge effect might have been influenced by the presence of hedgerows. The hedgerows could be a barrier to wind and dispersal (e.g., Lewis and Dibley 1970; Pasek 1988) and/or might cause shading on the field edges, which could affect beetle behavior and distribution (Fleming 1972; Rowe and Potter 2000). In addition, hedgerows could provide alternative food plants, which might also affect the behavior of beetles in the area, but no evidence was found that beetle damage in hedgerow plants was related to beetle density in adjacent soybeans. Unfortunately, because wind direction during this study was consistent (which is the usual case during the summer beetle flight season), the relative effects of wind and sun on beetle distribution near hedgerows were not able to be determined. Future studies, including possible experimental approaches, on the direction of beetle movement, and how host plants, aggregations, wind, and barriers affect their movement, would be of great benefit for determining the mechanism underlying the spatial patterns found in this study.

### Egg load

Egg loads increased significantly with increased transect distance from edge. Because beetle numbers also decreased significantly with increased transect distance from edge, the females in the center were more likely to be isolated from other beetles. Thus, these results are consistent with Regniere et al. (1983) and Kowles and Switzer (2012), who found evidence that females in aggregation areas had lower egg loads than females found away from aggregations. Fecund females may be looking for oviposition sites away from aggregations, or perhaps have moved away from an aggregation because they have no need to mate (Kowles and Switzer 2012). In contrast, females with relatively low egg loads may have just oviposited, need to locate food quickly, and therefore use kairomones produced by an aggregation to find a suitable host where they can feed (Loughrin et al 1996; Kowles and Switzer 2012). These females may need to re-mate as well, and by settling in an aggregation they can increase their likelihood of mating (Kowles and Switzer 2012).

Alternatively, this spatial distribution in egg load could have been due to the spatial distribution of suitable oviposition substrate. This possibility would predict that the females on the edge were closer to oviposition sites in surrounding habitat, and thus were more likely to have laid eggs recently. *P. japonica* do lay eggs in row-crop fields (Van Timmeren et al. 2000), but neither the likelihood of oviposition in these various sites, nor the movement behavior between the oviposition sites and feeding sites, are currently understood well enough to test this prediction.

### Potential management implications

The patterns in *P. japonica* distribution observed in this study indicate several factors may be influencing the movement and distribution of beetles. Higher densities of beetles occurred on field edges downwind. In particular, downwind edges adjacent to a hedgerow had high beetle densities. These patterns have important management implications for *P. japonica*. Because higher densities occured on field edges, control measures, such as insecticides, could be concentrated on field edges to reduce the amount applied. In addition, one could sample field edges to identify the presence of *P. japonica* without sampling large areas of the field. However, although looking for beetles on field edges could help identify beetle presence, the results of the present study indicate that those edge densities may not be representative of densities across the entire field, and thus care should be taken when inferring beetle impact on the whole field from numbers of beetles observed on edges. Finally, the patterns seen around hedgerows indicate windbreaks may have the potential to be a useful management tool, but future studies, under more varied conditions, would be necessary to make informed management decisions based on these patterns.
